# Presacral tumors: a retrospective analysis of 112 cases with emphasis on diagnostic challenges and surgical outcomes

**DOI:** 10.3389/fsurg.2025.1638820

**Published:** 2025-08-13

**Authors:** Shi Chen, Heng Deng, Ming Li, Xiaoli Fang

**Affiliations:** ^1^ Department of Anorectal Surgery, First Clinical Medical College, Anhui University of Chinese Medicine, Hefei, China; ^2^ Department of Anorectal Surgery, Second Affiliated Hospital, Anhui University of Chinese Medicine, Hefei, China; ^3^ Department of Anorectal Surgery, The First Affiliated Hospital of Anhui University of Chinese Medicine, Hefei, China

**Keywords:** presacral tumor, surgical approach, case series, tertiary referral center, diagnostic error burden

## Abstract

**Background:**

Presacral tumors are rare entities with heterogeneous pathology including malignant potential. Due to nonspecific symptoms mimicking common anorectal diseases, misdiagnosis remains a major challenge that may delay treatment and worsen prognosis, particularly for malignant variants. This study analyzes diagnostic pitfalls and surgical outcomes in a large single-center cohort.

**Methods:**

We retrospectively reviewed 112 presacral tumor cases treated at our tertiary colorectal center (2015–2025). Data included demographics, clinical presentation, misdiagnosis rates, imaging accuracy, surgical approaches, and complications. Statistical analysis utilized descriptive methods and Chi-square tests.

**Results:**

Among 112 patients (male 62, female 50; median age 52 years, range 18–93), 57% presented with anal/rectal pain, while 20.5% were asymptomatic. 85.7% of patients were referred from non-specialized centers. Misdiagnosis occurred in 29.5% (predominantly as anal fistula/abscess or pilonidal sinus). Preoperative imaging (MRI/CT) correctly diagnosed 60% of tumors >3 cm vs. 21.2% of smaller tumors (*P* < 0.001). Surgical approaches: 93.8% underwent transsacral/transanal resection, 6.2% required laparoscopic/combined abdominoperineal resection. Major complications (Clavien-Dindo grade III) occurred in 4.5% of patients (*n* = 5/112), including hemorrhage, rectal injury, and sacral nerve injury. No mortality occurred. Pathology revealed 11.6% malignancy risk.

**Conclusion:**

High misdiagnosis rates (29.5%) data support for heightened suspicion in patients with “refractory perianal sepsis”, especially given the potential for malignancy. MRI showed significantly higher diagnostic accuracy for tumors >3 cm. Transsacral/transanal resection is safe and effective for most cases (93.8%), with low major morbidity. Centralized management in specialized centers optimizes outcomes.

## 1 Introduction

Presacral tumors, arising from the retrorectal space—a complex embryological junction containing remnants of the neural tube, notochord, and hindgut—represent a rare yet clinically significant entity in colorectal surgery (1). With an estimated incidence of 1/40,000 (2), these lesions exhibit remarkable pathological diversity, encompassing over 25 subtypes ranging from benign cysts (e.g., tailgut cysts) to aggressive malignancies (e.g., chordomas or sarcomas) (3, 4). Critically, 10%–30% demonstrate malignant potential (5), underscoring the imperative for timely diagnosis and intervention.

Despite their clinical relevance, presacral tumors frequently elude early detection. Non-specific symptoms (e.g., perianal pain in 57% of our cohort) mimic common anorectal disorders such as anal fistulas or pilonidal disease, leading to misdiagnosis rates exceeding 30% in published series (6). This diagnostic delay carries profound implications: benign lesions may progress to cause irreversible nerve damage or fistulization, while malignant variants risk metastatic spread during prolonged diagnostic odysseys (7). Alarmingly, even at tertiary centers, over 25% of patients present following failed prior interventions for retrorectal tumors (8).

Surgical resection remains the cornerstone of cure, yet optimal approaches are debated. The choice between transsacral, transanal, or abdominal routes hinges on tumor size, location relative to S4, and suspicion of malignancy—factors poorly codified in current guidelines (9). Although minimally invasive techniques gain traction, their role in complex presacral pathology lacks robust evidence (10, 11). Compounding these gaps is the scarcity of large-scale analyses: most literature comprises case report or single-institution reports with <50 cases (12), insufficient to power clinical recommendations.

Leveraging one of Asia's largest presacral tumor cohorts (*n* = 112), this study aims to:

Quantify real-world misdiagnosis patterns and their impact on management timelines;

Evaluate imaging accuracy (MRI/CT) stratified by tumor size and pathology; Analyze surgical outcomes across approaches, emphasizing complications and recurrence; Propose a diagnostic algorithm integrating clinical red flags to mitigate delays, particularly for malignant cases. Our findings aspire to refine multidisciplinary paradigms for this orphan disease.

## 2 Materials and methods

### 2.1 Study design and setting

This retrospective cohort study adhered to the STROBE (Strengthening the Reporting of Observational Studies in Epidemiology) guidelines (13). We reviewed consecutive patients with histopathologically confirmed presacral tumors treated at a tertiary colorectal referral center (Department of Anorectal III, the First Clinical Medical College of Anhui University of Chinese Medicine) between January 2015 and December 2025. Ethical approval was obtained from the Institutional Review Board (No. AH012-2025), with waiver of informed consent for anonymized data.

### 2.2 Participants

**Figure 1:**
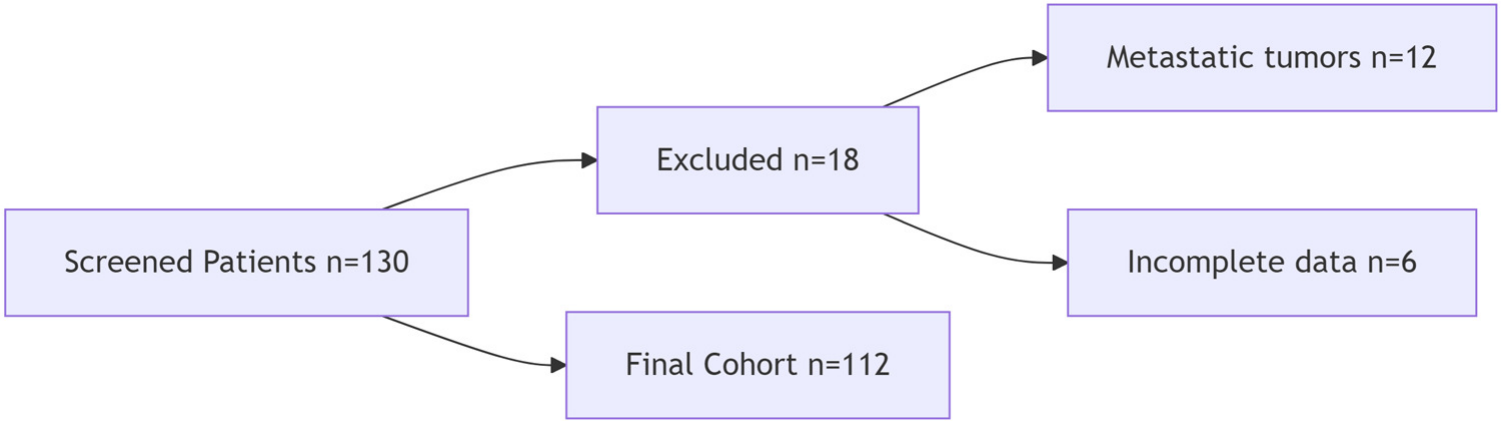
Patient selection flowchart (STROBE-compliant).

We included patients ≥18 years with histopathologically confirmed primary presacral tumors undergoing complete resection. Exclusion criteria were metastatic tumors and incomplete records (Figure 1: STROBE patient selection flowchart).

### 2.3 Data sources and variables

Clinical and operative data were extracted from structured electronic health records (EHR) using standardized protocols. Demographic data (age, sex, referral source) were extracted from registration records, while symptom duration and prior misdiagnoses were documented from clinic notes and referral letters. Tumor size (largest axial diameter) and radiologic suspicion of malignancy (irregular borders, diffusion restriction, or contrast enhancement) were assessed via blinded review of MRI/CT reports. Surgical approach (transsacral/transanal/abdominal) and complications were extracted from operative notes. Tumor type and malignancy confirmation (Ki-67 > 20% or lymphovascular invasion) were based on histopathology reports. 30-day morbidity (Clavien-Dindo classification) and recurrence were recorded from follow-up clinic visits.

### 2.4 Imaging protocols

MRI examinations were performed using 3 T scanners (Philips Ingenia) with standard pelvic sequences including T1/T2-weighted imaging and diffusion-weighted imaging, with tumor malignancy suspected when demonstrating irregular borders, diffusion restriction (ADC <1.0 × 10^−3^ mm^2^/s), or contrast enhancement. Complementary contrast-enhanced CT scans were acquired at 120 kVp with 2 mm slice thickness to evaluate potential bone erosion or lymphadenopathy, providing additional anatomical assessment of tumor extent and local invasion.

### 2.5 Bias mitigation

To minimize potential biases, we implemented multiple safeguards throughout the study. Misclassification bias was addressed through strict pathology confirmation protocols requiring all slides to be independently re-reviewed by specialized pathologists. Selection bias was mitigated by consecutively enrolling all eligible cases. For measurement bias control, digital rectal examination findings were standardized and performed exclusively by senior surgeons with ≥10 years of clinical experience in pelvic tumor assessment.

### 2.6 Statistical analysis

Statistical analyses were performed using R version 4.1.0 (packages: stats, epiR) under the guidance of Jun Zhang, PhD from Anhui University of Chinese Medicine. Continuous variables, after confirmation of non-normality through Shapiro–Wilk testing, were reported as medians with interquartile ranges [IQR], while categorical data were presented as counts (%). For group comparisons, Fisher's exact test was employed for categorical variables with expected counts below 5, chi-square tests for larger contingency tables, and Mann–Whitney *U*-tests for non-normally distributed continuous variables. Diagnostic accuracy metrics including sensitivity and specificity were calculated alongside odds ratios with 95% confidence intervals to evaluate imaging performance. All tests were two-tailed (*α*=0.05). No imputation was performed given <5% missingness (complete-case analysis).

## 3 Results

### 3.1 Baseline characteristics and clinical findings

The cohort comprised 112 patients with a median age of 52 years (range 18–93), showing a male predominance (55.4%, *n* = 62). Notably, 85.7% (*n* = 96) of patients were referred from non-specialized centers, underscoring the need for centralized management of these rare tumors. The median tumor diameter was 4.2 cm (range 0.5–20 cm), with 46.4% (*n* = 52) measuring ≤3 cm. Interestingly, 20.5% (*n* = 23) of tumors were discovered incidentally during evaluation for unrelated conditions (Table 1).

**Table 1:** Baseline characteristics of presacral tumor cohort [*n* = 112].

Variable	Value
Age (years)	52 [18–93]
Sex (Male:Female)	62:50
Referral status	
Local	16 (14.3%)
Regional referral	96 (85.7%)
Tumor size	
≤3 cm	52 (46.4%)
>3 cm	60 (53.6%)
Incidental finding	23 (20.5%)
Surgical approaches	
Transsacral	78.6% (*n* = 88)
Transanal	15.2% (*n* = 17)
Laparoscopic/combined	6.2% (*n* = 7)

### 3.2 Diagnostic performance

**Figure 2:**
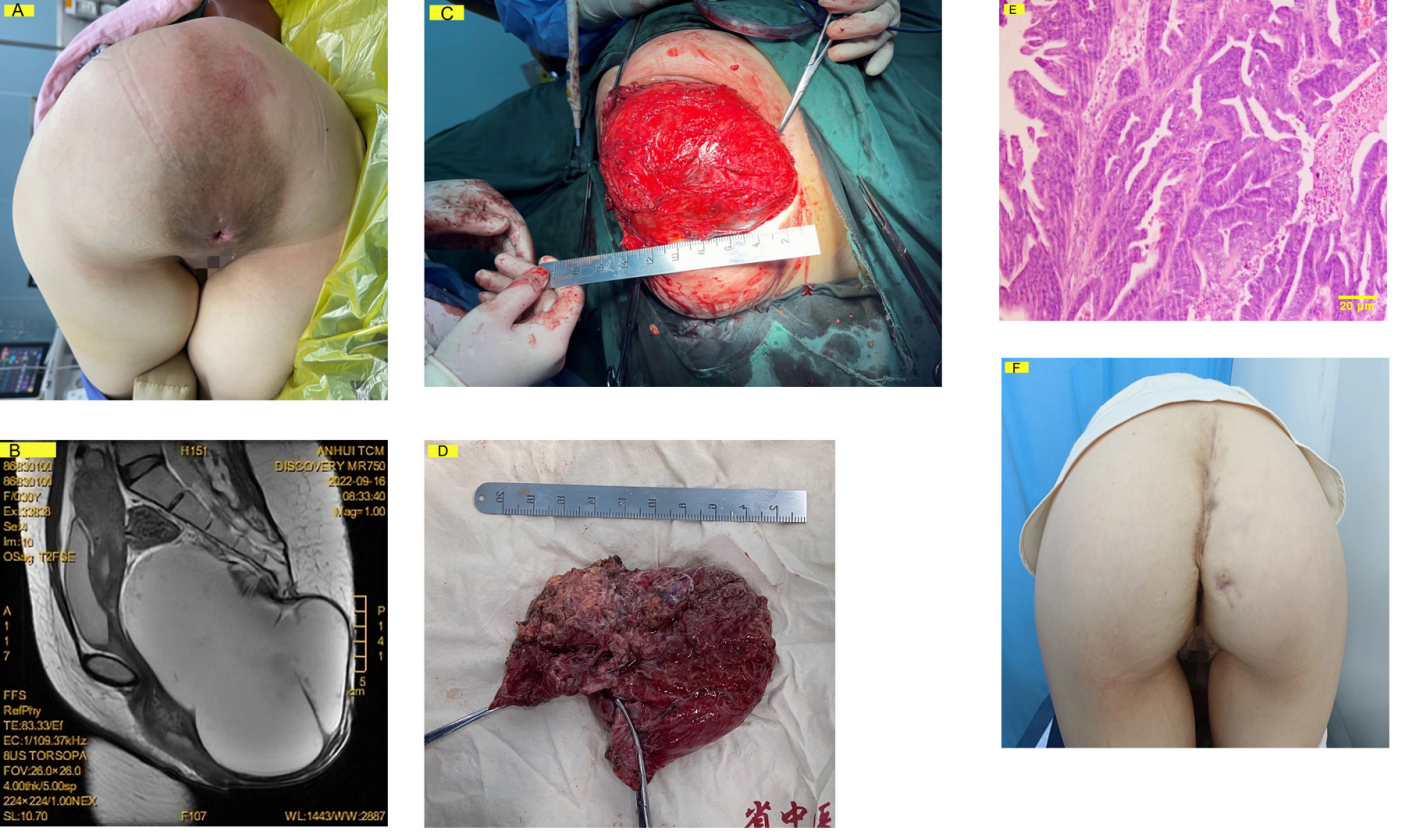
A 31-year-old female with an asymptomatic 20 × 15 cm presacral mass discovered incidentally and monitored for 2 years. **(A)** Preoperative clinical photograph (knee-chest position) demonstrating a 20 × 15 cm sacrococcygeal mass with normal overlying skin, firm-elastic consistency, and no tenderness. **(B)** Preoperative sagittal T2-weighted MRI revealing a multiloculated cystic-solid mass (maximum diameter: 18.5 cm) causing significant rectal compression. **(C)** Intraoperative view via sacral approach showing tumor exposure following coccygectomy (forceps indicate sacral resection margin). **(D)** Gross specimen (18.5 × 12.5 × 6.2 cm) with predominant solid component (dotted line). **(E)** Histopathology showed diagnostic uncertainty between malignant teratoma and primary adenocarcinoma, resolved through expert consultation at our referral center. **(F)** Postoperative 6-month follow-up demonstrating preserved gluteal fold symmetry after reconstruction (patient consent obtained). Perineal region obscured for privacy.

Digital rectal examination demonstrated high sensitivity (91.1%, 102/112) for detecting presacral masses. Prior to referral, 29.5% (*n* = 33) of cases had been misdiagnosed, most commonly as anal fistula (45.5%), pilonidal sinus (30.3%), or abscess (24.2%). Imaging accuracy showed significant variation by tumor size, with correct preoperative diagnosis achieved in only 21.2% of tumors ≤3 cm compared to 60% of larger tumors (*P* < 0.001, Table 2). Among giant tumors (>15 cm, *n* = 7), preoperative MRI accurately predicted resectability in all cases, with histopathological findings revealing unexpected malignant transformation in 2 cases (28.6%). One such case with diagnostic challenges between teratoma and primary adenocarcinoma is illustrated in Figure 2.

**Table 2:** Imaging limitations by tumor size.

Size group	Preoperative imaging accuracy	Odds ratio (95% CI)
>3 cm	60.0% (36/60)	4.82 (2.11–11.02)
≤3 cm	21.2% (11/52)	1.00 (Reference)
*P*-value	<0.001	—

Odds ratio calculated by logistic regression with ≤3 cm group as reference. Accuracy defined as correct preoperative imaging diagnoses per size group.

### 3.3 Surgical outcomes

Transsacral resection was performed in 78.6% of cases (*n* = 88), with transanal (15.2%, *n* = 17) and laparoscopic/combined approaches (6.2%, *n* = 7) reserved for selected cases (Table 3). Major complications (Clavien-Dindo grade III) occurred in 4.5% of patients (*n* = 5/112), including three cases of hemorrhage controlled with intraoperative hemostatic matrix (*n* = 2) or surgical packing with reoperation (*n* = 1), one rectal injury (≤5 mm defect) that was primarily repaired during the procedure without diversion and managed with 5-day bowel rest followed by contrast enema confirmation, and one case of sacral nerve injury requiring postoperative neurorehabilitation. Minor complications occurred in 12.5% (*n* = 14), primarily superficial wound infections treated with local care. With no mortality observed and a median follow-up of 36 months, the recurrence rate was 1.8% (*n* = 2/112), both occurring in benign cystic lesions (tailgut cysts) due to incomplete epithelial removal. No malignant cases recurred, likely attributed to R0 resection ± adjuvant therapy.

**Table 3:** Surgical approaches and outcomes (median follow-up: 36 months).

Approach	Cases (*n* = 112)	Ostomy rate	Drain use	Bowel prep	Additional maneuvers	Major complications
Transsacral	88 (78.6%)	0%	100%	20%	Coccygectomy: 52.3% (*n* = 46)	Hemorrhage: 3.4% (*n* = 3)
					Gluteal reconstruction: 3.4% (*n* = 3)	
Transanal	17 (15.2%)	0%	30%	100%	None	Rectal injury: 5.9% (*n* = 1);
Laparoscopic	7 (6.2%)	2 (28.6%)*	100%	100%	None	Nerve Injury: 14.3% (*n* = 1)

*Footnotes: Both ostomies were temporary (diverting ileostomies for rectal injury repair).

Major complications management: Hemorrhage (*n* = 3): Hemostatic matrix (*n* = 2), reoperation (*n* = 1); Rectal injury (*n* = 1): Primary repair, no diversion; Nerve injury (*n* = 1): Neurorehabilitation.”.

#### 3.3.4 Pathologic subgroup analysis

Malignant tumors showed distinct management patterns compared to benign cases. Among the 13 malignant tumors, 5 required laparoscopic or combined surgical approaches. The treatment strategy for benign tumors was predominantly transsacral or transanal resection (97/99), with only 2 cases requiring laparoscopic/combined approaches. Adjuvant therapy was administered in 7 of the 13 malignant cases. Diagnostic challenges were more pronounced in malignant tumors, with 38.5% (5/13) receiving initial misdiagnosis vs. 28.3% (28/99) of benign cases.

## 4 Discussion

Our study of 112 presacral tumors highlights persistent diagnostic delays, with 29.5% of cases initially misdiagnosed as benign anorectal disorders—a finding consistent with prior reports (25%–40%) (14). The asymptomatic nature of 20.5% of tumors emerged as a significant contributor to delayed detection (15). This contrasts with symptomatic presentations where pain typically prompts timely imaging. Digital rectal examination (DRE) demonstrated particular value, detecting 91.1% of masses, reinforcing its underutilized role in primary care screening (16): Digital rectal exam is the cheapest and most effective screening tool for presacral pathology.

In our cohort, stratification by tumor diameter revealed a significant detection threshold at 3 cm, with diagnostic accuracy markedly lower for tumors ≤3 cm (21.2%) compared to larger tumors (60.0%; OR = 4.82, 95%CI 2.11–11.02, *P* < 0.001). MRI's limited resolution for minute lesions (<2 cm) and radiologists’ unfamiliarity with rare presacral variants compound this (17). Moreover, the tumor diameters of our malignant cases are all greater than this threshold. Prior studies indicate tumors >3 cm are more likely to exhibit malignant features (5). Our data reinforce proposal: Pelvic MRI for atypical fistulas (failed >1 surgery), Non-palpable masses with persistent symptoms, any presacral lesion >2 cm on digital rectal exam.

The transsacral approach emerged as the predominant surgical strategy (78.6% of cases), demonstrating both safety (4.5% major morbidity) and versatility across diverse pathologies. Our utilization rate exceeds historical series (40%–70%) (9, 18), reflecting both technical refinements (e.g., coccygectomy in 41.1% of cases) and careful patient selection. For complex malignancies, laparoscopic/combined approaches proved valuable, particularly for hemorrhage control (19). Technical improvements include coccygectomy and gluteal reconstruction reduced recurrence and functional deficits, addressing historic concerns of poor wound healing (20). Technical Recommendations from High-Volume Experience: First, Incision planning: For transsacral resection, a curvilinear incision 2 cm lateral to the coccyx avoids midline wound tension; Second, Drain placement: Closed suction drains were routinely used for tumors >5 cm; Third, Bowel preparation: Limited to cases with suspected rectal invasion (15%), aligning with enhanced recovery protocols. Similar to the findings by Menteş et al. in a Turkish cohort (21).

**Figure 3:**
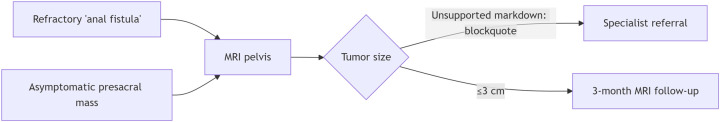
Diagnostic algorithm for suspected presacral tumors.

Diagnostic Red Flags (Figure 3): The high misdiagnosis rate our data support the use of pelvic MRI in cases of refractory perianal sepsis, even without palpable masses. Our proposed 3-cm imaging threshold mandates closer surveillance for small tumors. Centralized Care Model: 85.7% of patients were referred from underserved areas, referral rate confirms regional demand for specialized management, echoing NCCN guidelines for rare tumors (22), consistent with global disparities in pelvic tumor care (8). Our experience highlights the diagnostic challenges posed by presacral tumors’ embryological complexity (Table 4), exemplified by the malignant teratoma case where immunohistochemical markers (CDX2+/CK20+) failed to resolve the pathological dichotomy between malignant teratoma transformation (23), and tailgut origin theories (24). This underscores our recommendation for mandatory secondary pathological review in malignant cases.

**Table 4:** Histopathological diagnoses and malignancy rates.

Pathological diagnosis	Total cases (*n* = 112)	Malignant cases (%)*
Neuroendocrine tumors	36	5 (13.9%)
Gastrointestinal stromal tumors (GIST)	24	3 (12.5%)
Epidermoid cysts	19	0 (0%)
Tailgut cysts	12	0 (0%)
Chordomas	2	2 (100%)
Teratomas	2	1 (50%)
Hamartomas	8	0 (0%)
Schwannomas	1	0 (0%)
Neurofibromas	1	0 (0%)
Fibromas	5	0 (0%)
Lymphomas	2	2 (100%)
Total	112	13 (11.6%)

*Malignant transformation confirmed by Ki-67 > 20% and lymphovascular invasion.

Although, this study benefits from its large cohort of 112 cases. However, the retrospective design introduces potential selection bias, and while the high referral rate partially offsets the single-center limitation, the findings may not be fully generalizable. Additionally, the median 36-month follow-up period restricts assessment of long-term outcomes for malignant subtypes, suggesting need for extended surveillance in future studies. Future work should establish multicenter registries for malignant variants, explore PET-CT's role in ≤3 cm tumors, compare robotic vs. open approaches for complex cases.

## 5 Conclusions

This large-scale analysis of 112 presacral tumors establishes three critical practice-changing conclusions: First, the persistent misdiagnosis rate and atypical fistulas mandates routine pelvic MRI for refractory perianal cases, particularly given the 11.6% malignancy risk. Second, the transsacral approach demonstrated both dominance (78.6% utilization) and safety (4.5% major morbidity), confirming its role as the first-line strategy for presacral Tumors. Most significantly, our 85.7% referral rate and diagnostic challenges underscore that they require centralized multidisciplinary team management to optimize outcomes, reinforcing the need for regional referral networks in managing these complex tumors.

## Data Availability

The datasets presented in this study can be found in online repositories. The names of the repository/repositories and accession number(s) can be found below: Data Availability Statement: https://www.jianguoyun.com/p/DX8Q-X0Ql7C8DRjx6_sFIAA.
